# Loss of Pten promotes angiogenesis and enhanced *vegfaa* expression in zebrafish

**DOI:** 10.1242/dmm.012377

**Published:** 2013-05-29

**Authors:** Suma Choorapoikayil, Bart Weijts, Rianne Kers, Alain de Bruin, Jeroen den Hertog

**Affiliations:** 1 Hubrecht Institute-KNAW and University Medical Center, Utrecht, The Netherlands; 2 Department of Pathobiology, Faculty of Veterinary Medicine, Utrecht University, Utrecht, The Netherlands; 3 Institute of Biology, Leiden, The Netherlands

## Abstract

Angiogenesis, the emergence of vessels from an existing vascular network, is pathologically associated with tumor progression and is of great interest for therapeutic intervention. PTEN is a frequently mutated tumor suppressor and has been linked to the progression of many types of tumors, including hemangiosarcomas in zebrafish. Here, we report that mutant zebrafish embryos lacking functional Pten exhibit enhanced angiogenesis, accompanied by elevated levels of phosphorylated Akt (pAkt). Inhibition of phosphoinositide 3-kinase (PI3K) by LY294002 treatment and application of sunitinib, a widely used anti-angiogenic compound, suppressed enhanced angiogenesis in *Pten* mutants. Vegfaa has a crucial role in angiogenesis and *vegfaa* expression was upregulated in embryos lacking functional Pten. Interestingly, *vegfaa* expression was also upregulated in hemangiosarcomas from haploinsufficient adult zebrafish *Pten* mutants. Elevated *vegfaa* expression in mutant embryos lacking functional Pten was suppressed by LY294002. Surprisingly, sunitinib treatment dramatically enhanced *vegfaa* expression in *Pten* mutant embryos, which might account for tumor relapse in human patients who are treated with sunitinib. Combined treatment with suboptimal concentrations of sunitinib and LY294002 rescued enhanced angiogenesis in *pten* mutant embryos without the dramatic increase in *vegfaa* expression, suggesting a new approach for therapeutic intervention in VEGFR-signaling-dependent tumors.

## INTRODUCTION

*PTEN* is one of the most frequently mutated tumor suppressor genes found in cancer (Stokoe, 2001). Somatic deletion of *PTEN* leads to tissue-specific tumor formation and germline deletion of *PTEN* is associated with syndromes such as Cowden’s disease, Bannayan-Zonana and Lhermitte-Duclos disease (Liaw et al., 1997; Marsh et al., 1997; Zhou et al., 2003). Individuals with those syndromes share pathological features, including the formation of benign tumors and enhanced susceptibility to malignant cancer. PTEN, a lipid and protein phosphatase, antagonizes the phosphoinositide 3-kinase (PI3K)-Akt (also called PKB) pathway by balancing the cellular phosphatidylinositol (3,4,5)-trisphosphate [PtdIns(3,4,5)*P*_3_; also known as PIP3] level (Maehama and Dixon, 1998; Myers et al., 1998). Loss of PTEN increases PIP3 levels, resulting in constitutive activation of Akt signaling. Cell survival and proliferation are linked to activated Akt and thus uncontrolled activation of Akt leads to enhanced cell survival and proliferation, the hallmarks of cancer.

The zebrafish genome encodes two pten genes, designated *ptena* and *ptenb* (Croushore et al., 2005; Faucherre et al., 2008). Single mutants are viable and fertile, suggesting redundant function during development. Concomitant loss of Ptena and Ptenb results in embryonic lethality (Faucherre et al., 2008), reminiscent of loss-of-function of PTEN in mice (Di Cristofano et al., 1998), *Caenorhabditis elegans* (Mihaylova et al., 1999) and *Drosophila* (Goberdhan et al., 1999). We recently reported that haploinsufficiency of Pten in zebrafish (*ptena^+/−^ptenb^−/−^* and *ptena^−/−^ptenb^+/−^*) results in hemangiosarcoma formation during adult life (Choorapoikayil et al., 2012). The mechanism underlying uncontrolled endothelial growth resulting in hemangiosarcoma is not understood.

*In vitro* studies showed that inhibition of endogenous PTEN in cultured endothelial cells enhances vascular endothelial growth factor (VEGF) signaling (Huang and Kontos, 2002). VEGFs, secreted ligands binding to VEGF receptors (VEGFRs), are key players in vasculogenesis and angiogenesis. VEGF signaling promotes proliferation and differentiation of endothelial cells. The human VEGF family consists of five related growth factors, VEGFA, VEGFB, VEGFC, VEGFD and PIGF (placental growth factor). From these five secreted ligands, VEGFA was shown to be the main factor during angiogenesis, functioning as a mitogen, acting specifically on endothelial cells (Koch et al., 2011). It has been demonstrated that VEGFB promotes fatty acid uptake in endothelial cells (Hagberg et al., 2010; Li et al., 2008) and the role of VEGFB during angiogenesis is not fully elucidated yet. VEGFC is, together with VEGFD, crucial for lymphangiogenesis and has a minor role in vasculogenesis and angiogenesis (Koch et al., 2011).

We set out to study the function of Pten in endothelial cells *in vivo*. To this end, we investigated angiogenesis during embryonic development in *ptena^−/−^ptenb^−/−^* mutants. Here we report that *ptena^−/−^ptenb^−/−^* mutants displayed ectopic vessel growth. Inhibition of PI3K signaling rescued hyperplasia of endothelial cells. Moreover, treatment of *ptena^−/−^ptenb^−/−^* mutants with sunitinib, an angiogenesis inhibitor that selectively inhibits receptor tyrosine kinases (RTKs), also rescued enhanced angiogenesis. We found that elevated overall phosphorylated Akt (pAkt) levels in embryos were suppressed by PI3K inhibitors, and to a lesser extent by sunitinib.

**TRANSLATIONAL IMPACT**

**Clinical issue**

The *PTEN* gene is the second most frequently mutated tumor suppressor gene in human cancer. The gene encodes PTEN, a lipid and protein phosphatase, loss of which is associated with enhanced cell survival and proliferation, both hallmarks of cancer. Previously, this group reported that zebrafish (which express two pten genes, *ptena* and *ptenb*) that retain only a single wild-type allele of *ptena* or *ptenb* develop hemangiosarcomas, tumors of endothelial origin. The mechanisms underlying uncontrolled endothelial cell growth in the development of hemangiosarcomas are unknown. However, *in vitro* studies have suggested that loss of endogenous PTEN augments vascular endothelial growth factor (VEGF) signaling, which is involved in angiogenesis and vasculogenesis. The role of PTEN in endothelial cells *in vivo* has not yet been examined.

**Results**

Here, the authors generated a zebrafish model lacking functional Pten to analyze the role of the protein in cancer and development. Zebrafish embryos lacking functional Pten (*ptena^−/–^ptenb^−/–^*) displayed increased angiogenesis. The authors showed that hypervascularization could be rescued by exogenous *ptena* RNA and also by a phosphoinositide 3-kinase (PI3K) inhibitor, LY294002, and an angiogenesis inhibitor, sunitinib. Sunitinib acts by inhibiting receptor tyrosine kinases, including angiogenesis-promoting receptors in the VEGF signaling pathway. The authors also report that Pten mutants display enhanced expression of *vegfaa*, a ligand of VEGF receptors (VEGFRs). Interestingly, enhanced *vegfaa* expression was also observed in hemangiosarcomas from *Pten* haploinsufficient adult mutants. In the embryos, *vegfaa* expression was suppressed by LY294002, but, surprisingly, sunitinib treatment dramatically enhanced *vegfaa* expression. However, combination treatment of *Pten* mutant zebrafish embryos with low concentrations of LY294002 and sunitinib fully rescued the hypervascularization phenotype without enhancing *vegfaa* expression.

**Implications and future directions**

These results indicate that angiogenesis and *vegfaa* expression are enhanced in *Pten* zebrafish mutants, which could have important implications for humans with tumors that lack functional PTEN. Sunitinib has been used to suppress angiogenesis in cancer patients; however, successful treatment is followed by severe relapse in some cases. An increase in the expression of the human homolog of *vegfaa* in response to sunitinib treatment might explain this relapse. Moreover, this work provides evidence that combined treatment with a PI3K inhibitor and sunitinib suppresses hypervascularization without enhancing *vegfaa* expression, suggesting a new approach for therapeutic intervention in VEGFR-signaling-dependent tumors such as hemangiosarcomas.

*Vegfaa* expression was upregulated in *ptena^−/−^ptenb^−/−^* mutants and inhibition of PI3K abolished upregulation of *vegfaa*. Surprisingly, *vegfaa* expression was dramatically upregulated by sunitinib treatment. Combining PI3K inhibitors and sunitinib cooperatively rescued hypervascularization in *ptena^−/−^ptenb^−/−^* zebrafish embryos, revealing a tentative therapeutic approach to combat neovascularization in cancer.

## RESULTS

### *ptena^−/−^ptenb^−/−^* mutants display enhanced angiogenesis

**Fig. 1.**
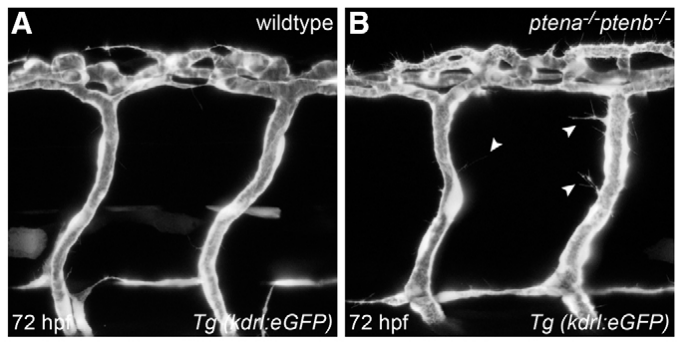
**Loss of Ptena and Ptenb leads to excessive filopodia formation in endothelial cells at 72 hpf.** Endothelial cells in living wild-type (A) and *ptena^−/−^ptenb^−/−^* mutant (B) embryos were visualized using *Tg(kdrl:eGFP)* and confocal imaging was performed at 70–72 hpf. Intersegmental vessels along the trunk in *ptena^−/−^ptenb^−/−^* mutants (4/4) show excessive filopodia formation (arrowheads), whereas no filopodia were observed in wild-type (0/4) embryos. Anterior to the left, 40× + 1.5 zoom, 0.5 μm step size.

**Fig. 2.**
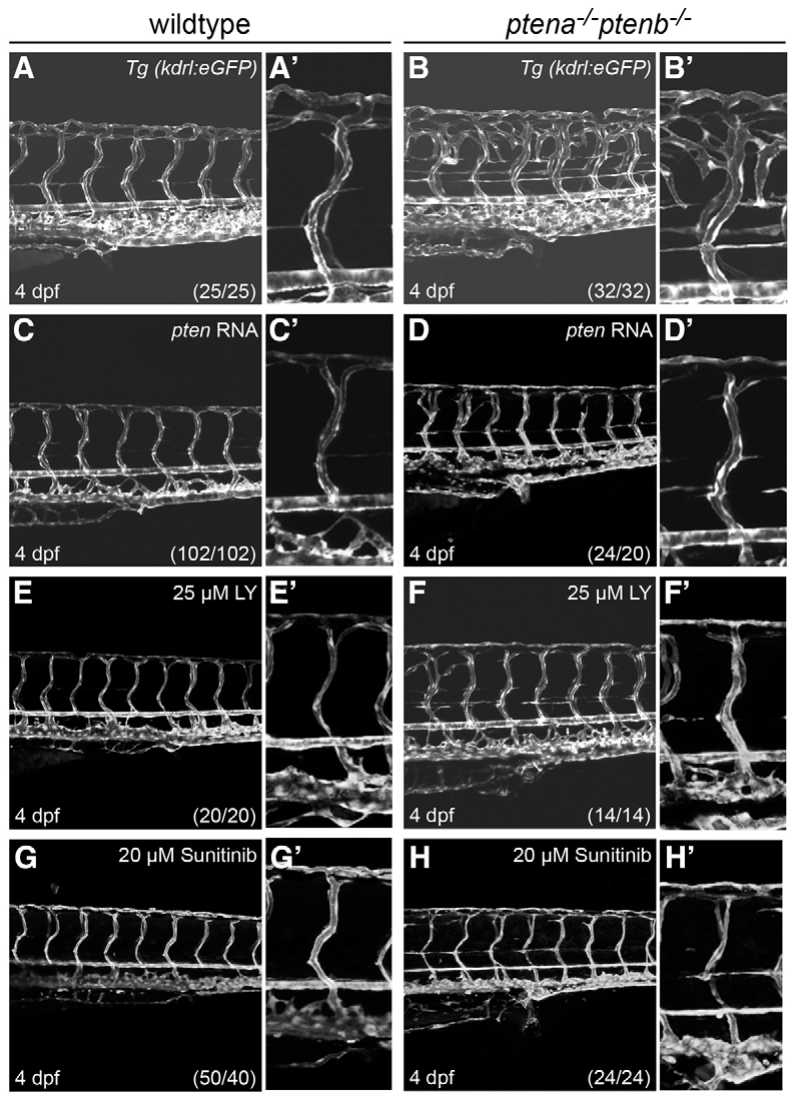
**Rescue of enhanced angiogenesis in *ptena^−/−^ptenb^−/−^* mutants by exogenous *ptena* mRNA, LY294002 or sunitinib.** The transgenic line, *Tg(kdrl:eGFP)*, was used to visualize the vasculature at 4 dpf in wild-type (A,C,E,G) and *ptena^−/−^ptenb^−/−^* (B,D,F,H) embryos. Close-ups of the intersegmental vessel above the urogenital opening are shown in adjacent panels. (A–B′) *ptena^−/−^ptenb^−/−^* mutants display ectopic vessel growth compared with wild-type embryos. (C–D′) 10 pg synthetic *ptena* mRNA was injected at the one-cell stage into wild-type and *ptena^−/−^ptenb^−/−^* embryos. (E–F′) 25 μM LY294002 (LY) was applied from 70–72 hpf onwards. (G–H′) 20 μM sunitinib was applied from 70–72 hpf onwards. Images were taken using a confocal microscope with 20×. The numbers in the bottom right corner represent the total number of embryos treated/the number of embryos showing the phenotype depicted here. Anterior to the left, 20×, 2 μm step size.

Haploinsufficiency of Pten leads to uncontrolled proliferation of endothelial cells, resulting in the formation of hemangiosarcomas in zebrafish (Choorapoikayil et al., 2012). To investigate how loss of Pten supports tumor growth and in particular how loss of Pten affects endothelial cells, we visualized the vasculature in zebrafish *ptena^−/−^ptenb^−/−^* mutant embryos using the *Tg(kdrl:eGFP)* line (Jin et al., 2005). The anatomy of the vasculature in the trunk was monitored between 2 and 4 dpf. We observed excessive sprouting of endothelial cells of the intersegmental vessels, in that these cells developed excessive filopodia from 72 hpf onwards (Fig. 1), resulting in ectopic vessel growth at 4 dpf (Fig. 2A–B′). Time-lapse imaging revealed that endothelial cells lacking Pten display protruding filopodia in a highly dynamic manner, whereas endothelial cells in siblings remain quiescent (supplementary material Movies 1, 2 and Fig. S1). Examination of three intersegmental vessels in 11 *ptena^−/−^ptenb^−/−^* mutant embryos at 4 dpf revealed that, on average, each intersegmental vessel formed two ectopic sprouts. At this stage, no sprouting was observed in the intersegmental vessels of siblings. Enhanced angiogenesis in *ptena^−/−^ptenb^−/−^* embryos was not restricted to the trunk and tail region, and was also observed in the head (supplementary material Fig. S2). Using confocal microscopy, we observed that newly formed vessels are perfused at 3 and 4 dpf (data not shown). Mutants retaining one wild-type allele (*ptena^+/−^ptenb^−/−^* or *ptena^−/−^ptenb^+/−^*) do not display any detectable malformations in vasculogenesis or angiogenesis during embryonic development (supplementary material Fig. S3). Taken together, we found that angiogenesis was enhanced in *ptena^−/−^ptenb^−/−^* mutants, resulting in hypervascularization.

### Hypervascularization in *ptena^−/−^ptenb^−/−^* mutants is rescued by LY294002 and sunitinib

To investigate the signaling underlying hypervascularization in *ptena^−/−^ptenb^−/−^* mutant embryos, we performed rescue experiments. Although vascularization throughout the embryo was affected, we focused on the trunk and tail region. Microinjection of *ptena* mRNA in *ptena^−/−^ptenb^−/−^* mutants at the one-cell stage suppressed the enhanced angiogenic phenotype at 4 dpf (Fig. 2D,D′). Similar rescues were obtained with microinjection of *ptenb* mRNA (data not shown). Ectopic expression of moderate amounts of Ptena in wild-type embryos did not affect the vasculature grossly (Fig. 2C,C′). The overall morphology of *ptena^−/−^ptenb^−/−^* mutant embryos is distinct from wild-type embryos in that the mutants are shorter and particularly the trunk and tail region is wider. These defects are largely, but not completely, rescued by injection of *ptena* mRNA (supplementary material Fig. S4). Morphological analysis revealed that, at 4 dpf, wild-type embryos injected with *ptena* mRNA displayed mild defects in body length (supplementary material Fig. S4A,C).

To investigate whether enhanced PI3K signaling is associated with enhanced angiogenesis in loss of Pten mutants, we treated embryos with the PI3K inhibitor LY294002 from the earliest time point at which we observed defects (70–72 hpf) onwards. Earlier treatment with LY294002 induces severe defects in the vasculature (Herbert et al., 2009) as well as defects as early as gastrulation (Montero et al., 2003). The overall morphology and vasculature of treated embryos was examined at 4 dpf. Wild-type embryos displayed mild defects in head size, and body length was reduced compared with non-treated embryos (supplementary material Fig. S4A,E). Consistent with our previous report (Faucherre et al., 2008), the morphological phenotype of *ptena^−/−^ptenb^−/−^* mutants was largely rescued by LY294002 treatment (supplementary material Fig. S4F,F′). In addition, the excessive sprouting phenotype in *ptena^−/−^ptenb^−/−^* mutants was largely rescued at 4 dpf after treatment with LY294002 (Fig. 2F,F′). Wild-type embryos treated with LY294002 displayed mild defects in vessel morphology, suggesting that endothelial cells are highly responsive to altered PI3K/Akt levels (Fig. 2E,E′). Thus, antagonizing the PI3K pathway suppressed ectopic vessel growth in *ptena^−/−^ptenb^−/−^* mutants, indicating that PI3K signaling has a central role in angiogenesis.

Next, we investigated whether inhibition of angiogenesis in *ptena^−/−^ptenb^−/−^* mutants suppressed the phenotype. To this end, we used the angiogenesis inhibitor sunitinib, which selectively inhibits RTKs (Roskoski, 2007), including VEGFRs in embryos. Wild-type embryos that were treated from 70–72 hpf onwards with sunitinib displayed no obvious morphological malformation in the vasculature (Fig. 2G,G′). Examination of the vasculature in *ptena^−/−^ptenb^−/−^* mutants at 4 dpf revealed that enhanced angiogenesis was suppressed by sunitinib treatment (Fig. 2G–H′). Our results suggest that signaling by sunitinib-sensitive RTKs has a crucial role in hypervascularization in Pten mutants.

### Elevated pAkt level in *ptena^−/−^ptenb^−/−^* mutants is suppressed by LY294002 and to a lesser extent by sunitinib

**Fig. 3.**
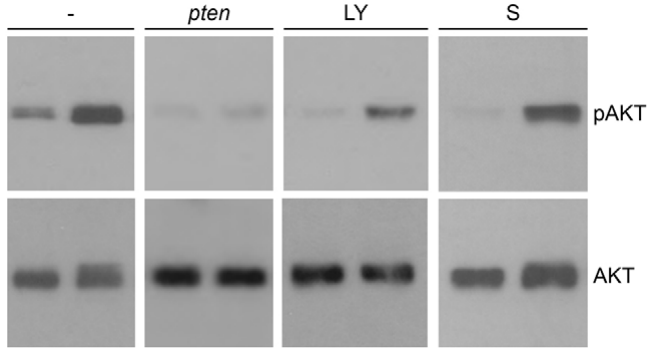
**Elevated pAkt level in *ptena^−/−^ptenb^−/−^* mutants is suppressed by LY294002 and to a lesser extent by sunitinib.** Wild-type (left lane of each blot) and *ptena^−/−^ptenb^−/−^* mutant (right lane of each blot) embryos were left untreated (–), were injected at the one-cell stage with *ptena* mRNA (*pten*), or were treated with 25 μM LY294002 (LY) or 20 μM sunitinib (S) from 72 hpf onwards. Single embryos were lysed at 4 dpf and the protein from individual embryos was isolated. The proteins were run on a denaturing SDS-polyacrylamide gel and transferred to PVDF membranes. After blocking, the blot was probed with phosphospecific anti-pAkt antibody (directed against pSer473), stripped and probed with Akt-specific antibody as a loading control. The number of individual embryos that were analyzed is: wild type, 24; mutant, 23; wild type + *pten*, 10; mutant + *pten*, 15; wild type + LY, 2; mutant + LY, 6; wild type + S, 5; mutant + S, 5. Representative blots are depicted here. The intensities of the bands were quantified (supplementary material Fig. S5).

Pten antagonizes PI3K signaling upstream of the Akt pathway and, consequently, loss of Pten leads to constitutive activation of Akt. We assessed pAkt levels by immunoblotting of individual embryos at 4 dpf. As expected, *ptena^−/−^ptenb^−/−^* mutants displayed dramatically enhanced levels of pAkt compared with wild-type embryos at 4 dpf (Fig. 3; supplementary material Fig. S5). Whereas pAkt levels varied from embryo to embryo, pAkt levels were consistently elevated in *ptena^−/−^ptenb^−/−^* embryos, compared with wild-type embryos. Re-expression of Ptena resulted in downregulation of elevated pAkt in *ptena^−/−^ptenb^−/−^* mutant embryos (Fig. 3). Similarly, we observed suppressed levels of pAkt in *ptena^−/−^ptenb^−/−^* mutant embryos upon treatment with the PI3K inhibitor LY294002 (Fig. 3). Sunitinib treatment reduced elevated pAkt levels in *ptena^−/−^ptenb^−/−^* mutants to a much lesser extent than did Ptena expression or LY294002 treatment (Fig. 3). pAkt levels were also reduced in wild-type embryos by expression of Ptena and by LY294002 or sunitinib treatment (Fig. 3). In summary, elevated pAkt levels in Pten mutants were suppressed by expression of Ptena and by treatment with LY294002 or sunitinib.

### *ptena^−/−^ptenb^−/−^* mutants display enhanced expression of *vegfaa*

**Fig. 4.**
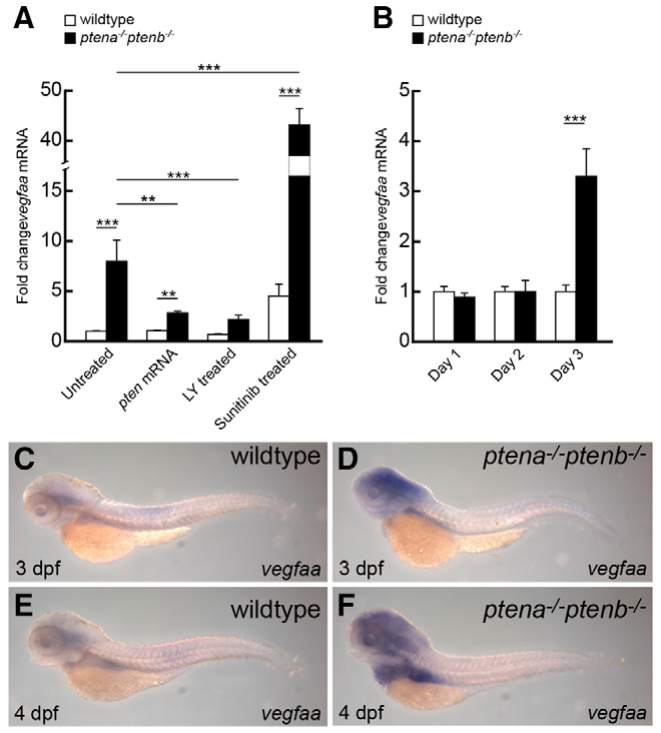
**Upregulated *vegfaa* expression in *ptena^−/−^ptenb^−/−^* mutants is diminished by LY294002 and enhanced by sunitinib.** (A,B) Quantitative PCR was performed to determine *vegfaa* expression levels in *ptena^−/−^ptenb^−/−^* mutants compared with wild type at 4 dpf (A) and at 1, 2 and 3 dpf (B). Rescue experiments were done by microinjection of *ptena* mRNA at the one-cell stage or by treatment with 25 μM LY294002 (LY) or 20 μM sunitinib from 72 hpf onwards. Wild-type control was set to 1 and all values were determined relative to the wild-type control at 3 dpf. Three embryos were pooled per condition and the data represent the results of at least two independent experiments. Statistical analysis (Kruskal-Wallis with Dunn’s post-hoc test) was performed using Excel and SPSS 20 (IBM); significance is indicated: **P<0.01; ***P<0.001. Note that the *y*-axis is discontinuous to accommodate the 40-fold increase in *vegfaa* expression upon sunitinib treatment of *ptena^−/−^ptenb^−/−^* mutants. (C–F) *In situ* hybridization was performed with a *vegfaa*-specific probe on 3-dpf or 4-dpf wild-type and *ptena^−/−^ptenb^−/−^* mutant embryos, as indicated. The number of embryos analyzed is: wild type 3 dpf, 6; wild type 4 dpf, 4; *ptena^−/−^ptenb^−/−^* 3 dpf, 4; *ptena^−/−^ptenb^−/−^* 4 dpf, 5. Representative pictures are depicted here. Pictures were taken with a 4.5× objective.

VEGF signaling, in particular that of *vegfaa*, is indispensable for angiogenesis. To address whether VEGF signaling is involved in enhanced angiogenesis in Pten mutants, we examined *vegfaa* expression levels at 4 dpf by quantitative PCR. *Vegfaa* expression was dramatically upregulated (eightfold) in *ptena^−/−^ptenb^−/−^* mutants compared with wild type (Fig. 4A). To assess at which developmental stage *vegfaa* expression is elevated in *ptena^−/−^ptenb^−/−^* mutants, we performed time course analysis at 1, 2 and 3 dpf. At 1 and 2 dpf of development, no difference in expression was detected between mutants and wild types. We found that *vegfaa* is significantly upregulated (threefold) from 3 dpf onwards (Fig. 4B), which coincides with the onset of enhanced filopodia formation in *ptena^−/−^ptenb^−/−^* mutant embryos (cf. Fig. 1). In order to verify upregulation of *vegfaa* expression, we performed whole-mount *in situ* hybridization. Consistent with the results obtained by quantitative PCR, we found elevated *vegfaa* expression at 3 and 4 dpf in mutants lacking Pten (Fig. 4C–F). The *vegfaa* expression pattern was rather diffuse and predominantly in the anterior region of the embryos. Next, we addressed whether the rescued angiogenic phenotype in *ptena^−/−^ptenb^−/−^* mutants after re-expression of Pten is associated with downregulation of *vegfaa*. We found that restoring Ptena expression in *ptena^−/−^ptenb^−/−^* mutants significantly downregulated the elevated *vegfaa* level (from eightfold to 2.5-fold) (Fig. 4A). Similarly, we found that *vegfaa* expression was significantly downregulated in *ptena^−/−^ptenb^−/−^* mutants by LY294002 (from eightfold to twofold; Fig. 4A). Surprisingly, *vegfaa* expression was dramatically enhanced by sunitinib in *ptena^−/−^ptenb^−/−^* mutants (from eightfold to 40-fold, compared with untreated wild type). In wild-type embryos, sunitinib treatment induced a modest increase in *vegfaa* expression (fourfold; Fig. 4A). Taken together, loss of Pten led to elevated *vegfaa* expression, which was rescued by inhibition of PI3K. Inhibition of angiogenesis using sunitinib greatly enhanced *vegfaa* expression in wild-type and *ptena^−/−^ptenb^−/−^* embryos, suggesting a feedback loop.

### Combined LY294002 and sunitinib treatment abolished enhanced *vegfaa* expression and reduced hypervascularization

**Fig. 5.**
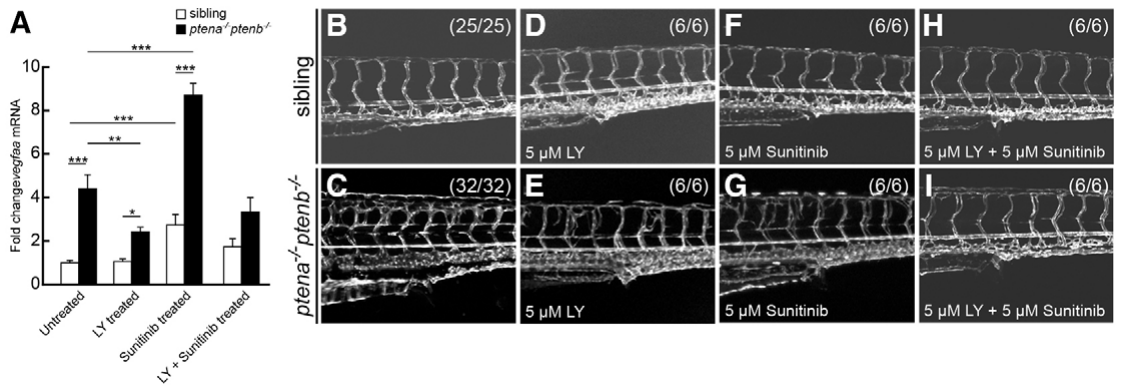
**Combined treatment with LY294002 and sunitinib rescued hypervascularization.** (A) Quantitative PCR was performed to determine *vegfaa* expression at 4 dpf in *ptena^−/−^ptenb^−/−^* mutants compared with siblings following treatment with suboptimal concentrations of LY294002 (LY, 5 μM) or sunitinib (5 μM), or both, from 72 hpf onwards. Three embryos per condition were pooled and used for quantitative PCR; at least two independent experiments were performed. Relative amounts were determined with wild type untreated set to 1.0. Statistical analysis (Kruskal-Wallis with Dunn’s post-hoc test) was performed using Excel and SPSS 20 (IBM); significance is indicated: **P<0.01; ***P<0.001. (B–I) Vasculature of wild-type and *ptena^−/−^ptenb^−/−^* embryos at 4 dpf was imaged in the *Tg(kdrl:eGFP)* line by confocal microscope with 20×. The embryos were treated with suboptimal concentrations of LY294002 (LY), sunitinib or both as indicated. Representative embryos are depicted; anterior to the left.

Sunitinib is a widely used anti-angiogenic compound that prevents neovascularization (Roskoski, 2007). Our results demonstrate that sunitinib treatment led to dramatic upregulation of *vegfaa* expression, particularly in *ptena^−/−^ptenb^−/−^* mutant embryos (Fig. 4). LY294002 treatment rescued elevated *vegfaa* expression to some extent. We hypothesized that LY294002 treatment might suppress sunitinib-induced *vegfaa* expression and the two inhibitors might cooperate to suppress enhanced angiogenesis. To test this, we combined LY294002 and sunitinib at suboptimal doses. A suboptimal concentration of LY294002 (5 μM) did not fully repress enhanced angiogenesis (Fig. 5B–E), but did suppress enhanced *vegfaa* expression in *ptena^−/−^ptenb^−/−^* mutant embryos (Fig. 5A), suggesting that *vegfaa* expression is tightly regulated by PI3K signaling. A suboptimal concentration of sunitinib (5 μM) did not fully repress enhanced angiogenesis in *ptena^−/−^ptenb^−/−^* mutant embryos (Fig. 5G) and still led to an eightfold increase in *vegfaa* expression (Fig. 5A), indicating that a slight modification of VEGFR signaling still has a dramatic effect on *vegfaa* expression. Concomitant application of suboptimal concentrations of LY294002 and sunitinib significantly suppressed *vegfaa* expression and fully inhibited hypervascularization in *ptena^−/−^ptenb^−/−^* mutant embryos (Fig. 5A,I). Analysis of pAkt levels following treatment with suboptimal concentrations of LY294002 or sunitinib indicated that these treatments did not fully suppress enhanced pAkt levels in *ptena^−/−^ptenb^−/−^* mutants. Combined treatment led to further downregulation of pAkt, but still did not completely suppress pAkt levels (supplementary material Fig. S6). Our data indicate that simultaneous partial inhibition of PI3K and VEGFR signaling cooperatively suppressed enhanced angiogenesis in *ptena^−/−^ptenb^−/−^* mutant embryos.

### Hemangiosarcoma formation in Pten haploinsufficient fish is accompanied by elevated *vegfaa* expression

**Fig. 6.**
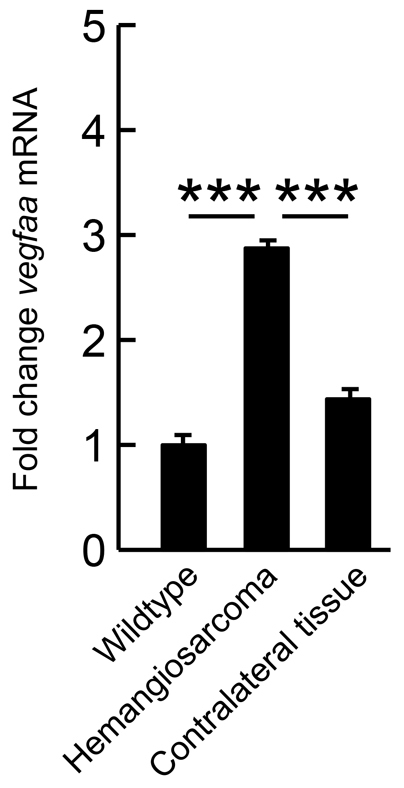
**Elevated expression of *vegfaa* in hemangiosarcoma.** Hemangiosarcoma tumor material of *ptena^+/−^ptenb^−/−^* mutants (*n*=3) was isolated. Contralateral tissue from the tumor-bearing fish and tissue from the same area in wild-type adult fish were isolated as control. RNA was isolated and quantitative PCR was performed to establish *vegfaa* expression. Statistical analysis was performed using Excel and fold-change values were determined with wild type set to 1.0; significance is indicated: ***P<0.001.

*Ptena^+/−^ptenb^−/−^* and *ptena^−/−^ptenb^+/−^* mutant adult fish are prone to develop hemangiosarcomas during their lifetime (Choorapoikayil et al., 2012). We have established that these hemangiosarcomas are preferentially formed in the rete mirabile, a highly vascularized tissue that is connected to the eye bulb. In general, hemangiosarcomas are associated with the vasculature and consist of perfused endothelial lumens. We investigated whether *vegfaa* expression was enhanced in hemangiosarcomas of *pten* mutant adult fish by quantitative PCR. We isolated RNA from the tumors and from contralateral tissue of the same animals and, as a control, we isolated RNA from roughly the same tissue in wild-type zebrafish. *Vegfaa* expression was threefold higher in the hemangiosarcoma than in wild-type tissue. *Vegfaa* expression in the contralateral tissue from the tumor-bearing fish was not significantly different from *vegfaa* expression in wild type (Fig. 6). Taken together, we show that *vegfaa* expression is enhanced in hemangiosarcomas, which might enhance tumor growth.

## DISCUSSION

PTEN is one of the most frequently mutated tumor suppressor genes in cancer. Concomitant loss of both *pten* genes in zebrafish leads to hyperplasia and dysplasia, resulting in embryonic lethality by 5 dpf (Faucherre et al., 2008). Mutants that retain one wild-type *pten* allele (*ptena^+/−^ptenb^−/−^* or *ptena^−/−^ptenb^+/−^*) are prone to develop endothelial-derived hemangiosarcomas later in life. Here, we investigated angiogenesis in the absence of functional Pten during zebrafish embryogenesis and found a dramatic hypervascularization in the vasculature throughout the embryo. Single *pten* mutants and mutants retaining one active *pten* allele (*ptena^+/−^ptenb^−/−^* or *ptena^−/−^ptenb^+/−^*) do not display any malformation in the vasculature. Hence, we conclude that Ptena and Ptenb have redundant functions in angiogenesis/vasculogenesis. In *ptena^−/−^ptenb^−/−^* mutants, we observed enhanced sprouting from 3 dpf onwards, resulting in the formation of ectopic blood vessels at 4 dpf. Normally, once the vasculature has been established, endothelial cells are quiescent and rarely form new branches. The absence of defects in vasculature at earlier time points in *ptena^−/−^ptenb^−/−^* embryos might be due to maternally provided Pten. However, this is unlikely because immunoblotting demonstrated that maternally contributed Pten was not detectable anymore from 60 hpf onwards (data not shown), well before the stage at which we observed enhanced angiogenesis. Perhaps Pten is not essential for vasculogenesis, i.e. *de novo* formation of blood vessels, and it only has a role in angiogenesis. Interestingly, it has been reported that PI3K signaling is essential for angiogenesis in mouse and fish development. Mouse mutant embryos with a homozygous mutation in the PI3K catalytic subunit (p110α^D933A/D933A^) show regular heartbeat and blood flow in central vessels until E10.5, indicating that vasculogenesis is normal. However, at E12.5, phosphorylation of Akt in p110α^D933A/D933A^ mutants is absent and embryos are lethal, exhibiting primary angiogenic remodeling defects (Graupera et al., 2008). We conclude that the loss of Pten induced defects in angiogenesis, not vasculogenesis.

Hypervascularization was not limited to the trunk area. We also observed massive increases in blood vessels in other areas of the embryo, including the head, by imaging *ptena^−/−^ptenb^−/−^* and wild-type embryos in the *Tg(kdrl:eGFP)* line (supplementary material Fig. S2). However, we focused on hypervascularization in the trunk and tail and investigated the molecular basis for upregulated endothelial proliferation in Pten mutant embryos by treatment with the inhibitors LY294002 and sunitinib. Treatment of *ptena^−/−^ptenb^−/−^* embryos with the PI3K inhibitor LY294002 from 72 hpf onwards rescued the hypervascularization phenotype at 4 dpf, indicating that these defects were caused by enhanced PI3KAkt signaling. Consistent with this notion is that elevated pAkt levels in Pten mutant embryos were suppressed by LY294002 treatment. The morphological defects in *ptena^−/−^ptenb^−/−^* mutants were also largely rescued by LY294002 treatment, which is consistent with our earlier report in which we treated embryos from 2 dpf onwards (Faucherre et al., 2008). Inhibition of PI3K at very early stages induced severe gastrulation defects (Montero et al., 2003), which precludes a full rescue of the loss of Pten phenotype by early treatment with LY294002.

Sunitinib treatment led to a full rescue of hypervascularization at 4 dpf. Yet, sunitinib did not fully suppress enhanced pAkt levels in *ptena^−/−^ptenb^−/−^* mutants. Sunitinib selectively inhibits a subset of RTKs, including the angiogenic VEGFR1, VEGFR2 and PDGFRβ (Roskoski, 2007). PI3K-Akt signaling downstream of other RTKs is not affected by sunitinib. Therefore, it is not surprising that sunitinib treatment did not fully suppress pAkt levels in *ptena^−/−^ptenb^−/−^* mutants. Apparently, inhibition of the angiogenic RTKs by sunitinib fully rescued hypervascularization in *ptena^−/−^ptenb^−/−^* mutants.

It seems that endothelial cells are particularly sensitive to loss of Pten. Previously, we reported that *pten* haploinsufficient zebrafish predominantly developed hemangiosarcomas, tumors of endothelial origin (Choorapoikayil et al., 2012). Moreover, recent work demonstrated that mouse endothelial cells lacking Pten are hypersensitive to vascular growth factor stimulation (Hamada et al., 2005). Enhanced sensitivity of endothelial cells to loss of Pten might be intrinsic to these cells. However, the finding that *vegfaa* expression is enhanced in *ptena^−/−^ptenb^−/−^* embryos suggests that this might contribute to enhanced sensitivity of endothelial cells to loss of Pten, because these cells express VEGFRs, providing positive feedback. Upregulation of VEGF expression in response to deletion of Pten is not unprecedented. siRNA-mediated knockdown of PTEN in a panel of pancreatic cell lines led to upregulation of VEGF expression (Ma et al., 2009). Moreover, ectopic expression of PTEN in the chronic myelogenous leukemia cell line, K562, led to reduced expression of VEGF (Zhiyong et al., 2012), which is consistent with our data in zebrafish. Elevated *vegfaa* expression in *ptena^−/−^ptenb^−/−^* zebrafish embryos is suppressed by treatment with LY294002, indicating that upregulation of *vegfaa* expression in *ptena^−/−^ptenb^−/−^* embryos is dependent on PI3K signaling. Sunitinib treatment led to a dramatic increase in *vegfaa* expression, particularly in *ptena^−/−^ptenb^−/−^* mutant embryos, suggesting a feedback mechanism. Inhibition of VEGFR1 and VEGFR2 and a subset of other RTKs enhanced expression of the VEGFR ligand Vegfaa. The mechanism underlying transcriptional regulation of *vegfaa* in Pten mutants and in response to inhibitors remains to be determined.

VEGF signaling is crucial for vascular development during embryogenesis. Elevated levels of *vegfaa* mRNA expression were detected from 72 hpf onwards, which is concomitant with the onset of enhanced angiogenesis, suggesting a causal relation. To address directly whether elevated *vegfaa* expression induced enhanced angiogenesis, we used morpholinos to knockdown Vegfaa expression. Unfortunately, Vegfaa knockdown induced massive defects in vasculature in wild-type embryos, consistent with previous reports (Nasevicius et al., 2000; Weijts et al., 2012), precluding assessment of the effect of Vegfaa knockdown on angiogenesis in *ptena^−/−^ptenb^−/−^* embryos. Elevated expression of *vegfaa* was not limited to *ptena^−/−^ptenb^−/−^* embryos. Significant upregulation of *vegfaa* expression was also observed in hemangiosarcomas that were isolated from adult zebrafish mutants that retained one wild-type allele of *pten*. Hemangiosarcomas are tumors that consist of endothelial cells and exhibit constitutive expression of Vegfr2 (*kdrl*) (Jinnin et al., 2008). Elevated *vegfaa* expression will result in a positive feedback loop, which might account for the hyperproliferation of endothelial cells in the hemangiosarcoma and hence contribute to tumor growth.

Sunitinib is commonly used as an anti-angiogenic drug to prevent (tumor) angiogenesis. Clinical reports describe cases in which, after administration of sunitinib, tumor relapse occurred with severe growth and increased metastatic behavior (Kikuchi et al., 2012; Tielen et al., 2012; Tonini et al., 2010). Here we discovered that treatment with sunitinib led to upregulation of *vegfaa* in wild-type embryos and to a further upregulation of *vegfaa* expression in mutant embryos lacking Pten. Transcriptional upregulation of *VEGFA* expression in response to sunitinib in patients will result in long-term enhanced VEGFA expression. By the time sunitinib has lost its potency, VEGFA expression is still elevated, leading to hyperactivation of VEGFRs, resulting in hyperproliferation of endothelial cells, hence explaining the tumor relapse after sunitinib treatment. Treatment with suboptimal concentrations of LY294002 and sunitinib did not lead to dramatic increases in *vegfaa* expression in zebrafish embryos, yet it fully rescued the hypervascularization phenotype. These results suggest that combined treatment might represent a novel approach for therapeutic intervention.

## MATERIALS AND METHODS

### Zebrafish husbandry

*ptena^−/−^ptenb^−/−^* and *Tg(kdrl:eGFP)* (Jin et al., 2005) were maintained, crossed, raised and staged as described (Kimmel et al., 1995; Westerfield, 2000). All procedures involving experimental animals were approved by the local animal experiments committee and performed in compliance with local animal welfare laws, guidelines and policies, according to national and European law.

### Immunoblotting

Single embryo lysates were obtained from wild type and *ptena^−/−^ptenb^−/−^* at 4 dpf using lysis buffer (50 mM HEPES, pH 7.4, 15 mM NaCl, 1 mM MgCl_2_, 10% glycerol, 1% Triton X-100, 1% sodium orthovanadate and protease inhibitors, including 5 mM beta glycerophosphate, 1 μg/ml aprotinin, 5 mM NaF, 1 mM Na_3_VO_4_ and 1 μg/ml leupeptin). Samples were mixed with 2× Laemmli sample buffer, boiled for 5 minutes and proteins were run on SDS-polyacrylamide gels. Immunoblotting was performed according to standard procedures, using p-Ser473-Akt (1:2000, Cell Signaling) and Akt (1:1000, Cell Signaling) antibodies.

### Confocal and brightfield microscopy

Fluorescence images of transgenic embryos were acquired using TCS-SPE and processed with ImageJ. Embryos were anesthetized with Tricaine and mounted on a glass cover dish with 0.7% low melting agarose and covered with standard E3 medium. Whole-mount brightfield images were taken with a Leica DC 300F stereomicroscope or a Zeiss Axioplan microscope connected to a Leica DFC480 camera.

### RNA isolation, cDNA synthesis and quantitative PCR

Total RNA was extracted using the RNeasy Mini Kit according to the manufacturer’s instructions (Qiagen). cDNA was synthesized with random hexamer primers according to the manufacturer’s instructions (Fermentas). Quantitative PCR was performed on a MyiQ cycler (Bio-Rad) using SYBRgreen chemistry (Bio-Rad). Three reference genes were used: tata box binding protein (*TBP*), elongation factor 1α (*EF1α*) and *β-actin*. Sequences of oligonucleotide are listed in Table 1. MIQE standards were applied to our protocols (Bustin et al., 2009). RNA extraction has been performed from three pooled embryos for each condition. For statistical analysis of two groups, unpaired *t*-test, or in case of unequal variances, Mann-Whitney *U*-test were used. For statistical analysis of multiple groups, 1-way ANOVA, or in case of unequal variances, Kruskal-Wallis test was used. Dunn’s post-hoc test was used to compare between selected groups. *P*-values <0.05 were considered significant. Statistical analysis was performed using SPSS 20 (IBM).

**Table 1.**
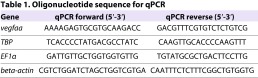
Oligonucleotide sequence for qPCR Gene

### LY294002 and sunitinib treatment, and *pten* RNA injection

Embryos were treated from 70–72 hpf onwards with 25 μM LY294002 (Calbiochem) or 20 μM sunitinib malate (Sigma), unless stated otherwise. Control embryos were mock treated with DMSO and the presence of *ptena^−/−^ptenb^−/−^* mutations was confirmed by genotyping as described. Embryos were kept in the dark during treatment. *ptena* and *ptenb* cDNA was cloned in pCS2+. 5′ capped sense RNA was synthesized using the mMessage mMachine kit from Ambion according to the manufacturer’s instructions and 10 pg/nl injected at the one-cell stage.

### *In situ* hybridization

Whole-mount *in situ* hybridization on 3-dpf and 4-dpf embryos was performed as described (Thisse and Thisse, 2008) using a *vegfaa*-specific probe (Liang et al., 2001).

## Supplementary Material

Supplementary Material
