# Supplementary material for: Loss of Pten promotes angiogenesis and enhanced vegfaa expression in zebrafish
Source: Dis Model Mech. 2013 May 29;6(5):1159–66. doi: 10.1242/dmm.012377 (PMC3759335; doi:10.1242/dmm.012377)
Supplement: Supplementary Material [file supp_6_5_1159__index.html]

Loss of Pten promotes angiogenesis and enhanced vegfaa expression in zebrafish — Supplementary Material 

# Loss of Pten promotes angiogenesis and enhanced *vegfaa* expression in zebrafish

## 

**Files in this Data Supplement:**

- **Supplementary Material PDF**
