# Supplementary material for: Loss of Pten promotes angiogenesis and enhanced vegfaa expression in zebrafish
Source: Dis Model Mech. 2013 May 29;6(5):1159–66. doi: 10.1242/dmm.012377 (PMC3759335; doi:10.1242/dmm.012377)
Supplement: Supplementary Material [file supp_012377_DMM012377.pdf]

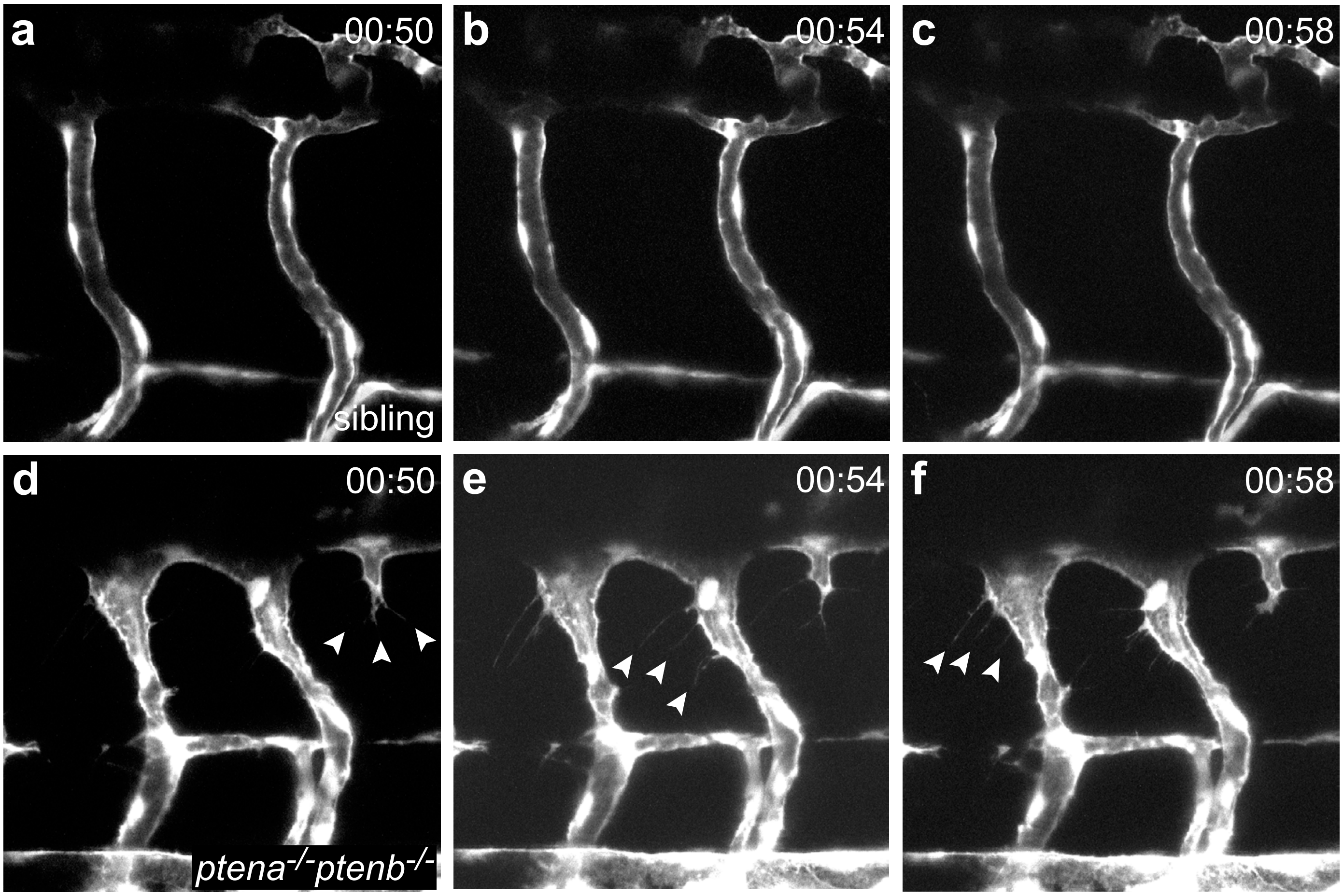

Suppl Figure 1. Endothelial cells lacking Pten display protruding filopodia from 75 hpf onwards.
Still frames from time lapse movies of *Tg(kdrl:eGFP)* sibling (Suppl Mov 1, panel a-c) and *ptena-/-ptenb-/-* mutant embryo (Suppl Mov 2, panel d-f) showing two intersegmental vessels in the trunk of the embryo. Endothelial cells in *ptena-/-ptenb-/-* mutants display filopodia formation (arrowheads), whereas endothelial cells in siblings do not. Images were taken every 2 minutes, maximum projections of z-stacks (1 µm step size) were used to generate the time lapse movies. Anterior to the left, 40x with 2 zoom, time is indicated in hours and minutes.

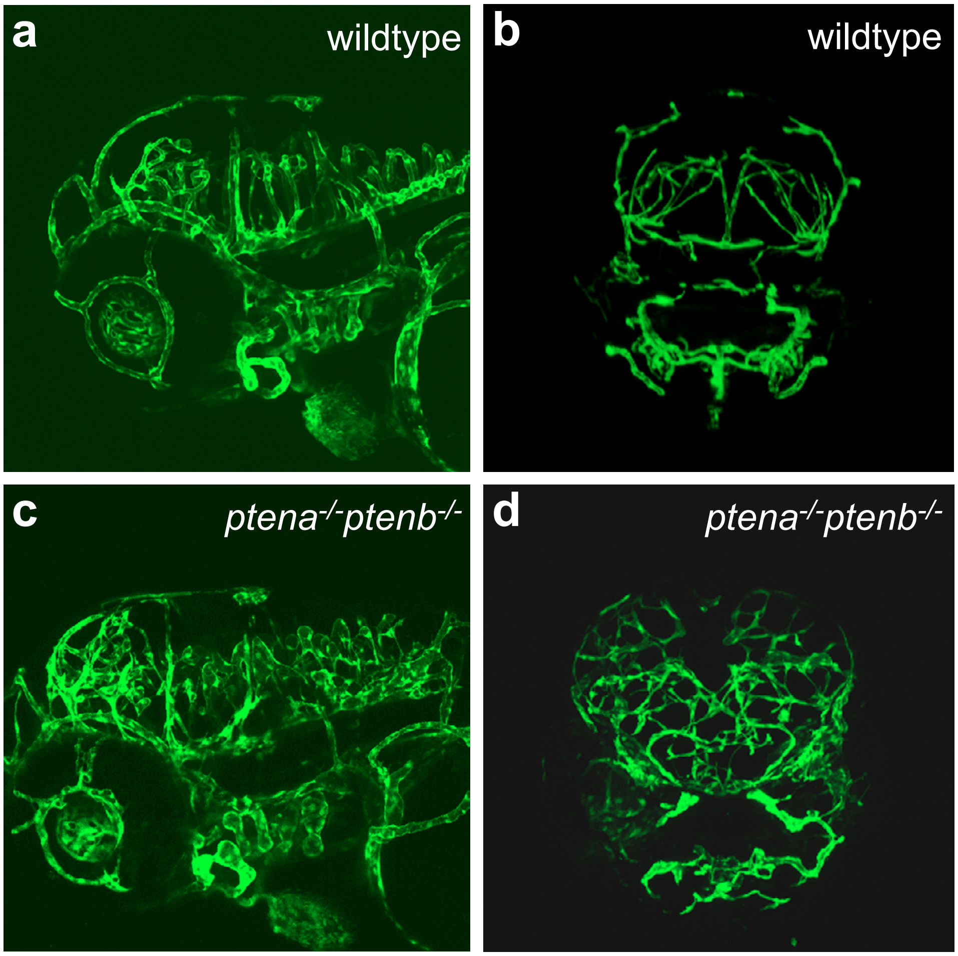

Suppl Figure 2. Enhanced angiogenesis in head vasculature in *ptena-/-ptenb-/-* mutant embryos
Endothelial cells in wildtype (a, b) and *ptena-/-ptenb-/-* mutants (c, d) were visualized using *Tg(kdrl:eGFP)* by confocal imaging at 4 dpf. Anterior to the left, 20x, 2 µm step size, lateral (a, c) and sagittal (b, d) view.

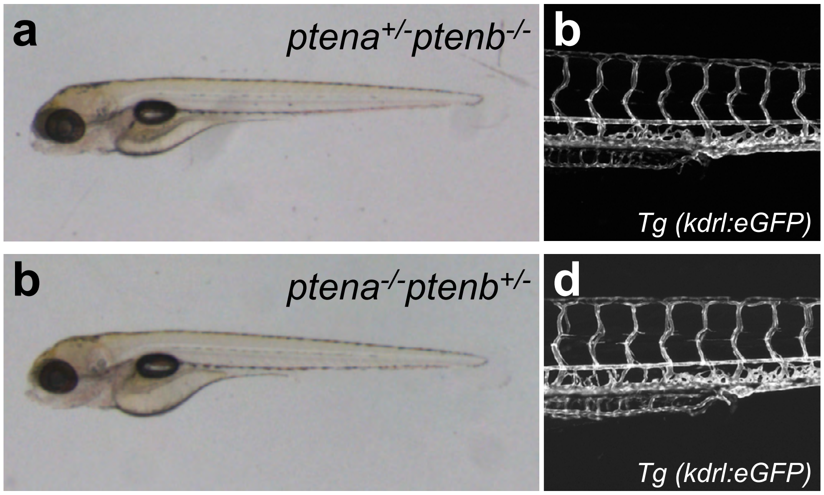

Suppl Figure 3. Morphology and vasculature are not perturbed in *ptena+-/-ptenb-/-* or *ptena-/-ptenb+/-* mutants.
The morphology (a, c) and vasculature (b, d) was visualized in *ptena+-/-ptenb-/-* (a,b) and *ptena-/-ptenb+/-* (c,d) mutants by (confocal) microscopy using *Tg(kdrl:eGFP)* at 4 dpf. No defects were observed in mutants. Anterior to the left, 20x, 2 µm step size.

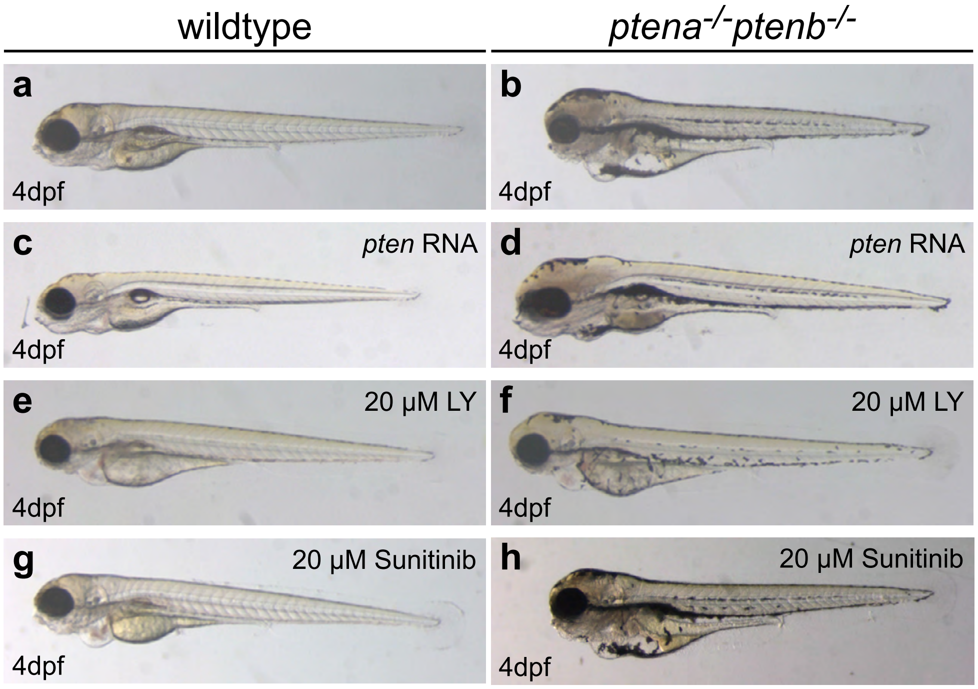

Suppl Figure 4. Rescue of morphological defects in *ptena-/-ptenb-/-* mutants by exogenous Ptena, LY294002 and Sunitinib.
At 4 dpf *ptena-/-ptenb-/-* mutants display severe morphological defects compared to wildtype (a, b): head and eye edema, wider set eyes, enlargement of yolk sac, reduced body length. Expression of exogenous Ptena did not cause malformation in development of wildtype embryo, but largely rescued the developmental defects in *ptena-/-ptenb-/-* mutants (c, d). Treatment of embryos from 72 hpf onwards with 25 µM LY294002 resulted in mild growth defects in the head region in wildtype embryos and almost complete rescue of developmental defects in *ptena-/-ptenb-/-* mutants (e,f). Treatment with 20 µM Sunitinib from 72 hpf onwards did not induce obvious morphological defects in wildtype embryos and largely rescued the morphological malformations in *ptena-/-ptenb-/-* mutants (g,h).

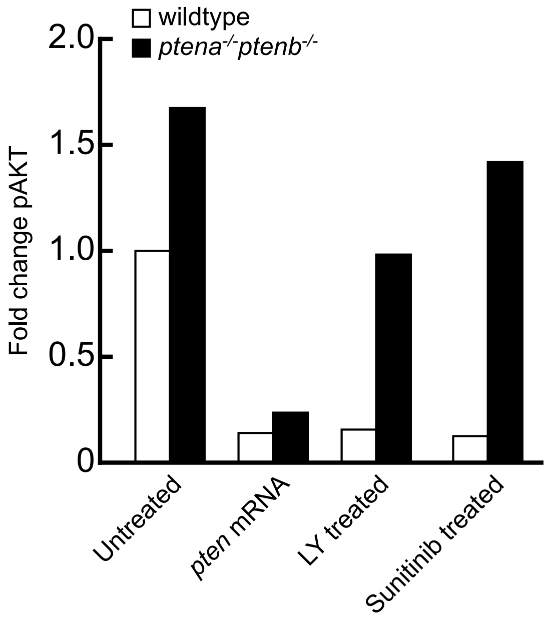

Suppl Figure 5. Quantification of pAkt levels in Fig. 3.
Relative intensities of bands on immunoblots from Fig. 3 were quantified using Image J software (http://rsbweb.nih.gov/ij/) with the gel analyzer tool. The fold change was calculated using Excel.

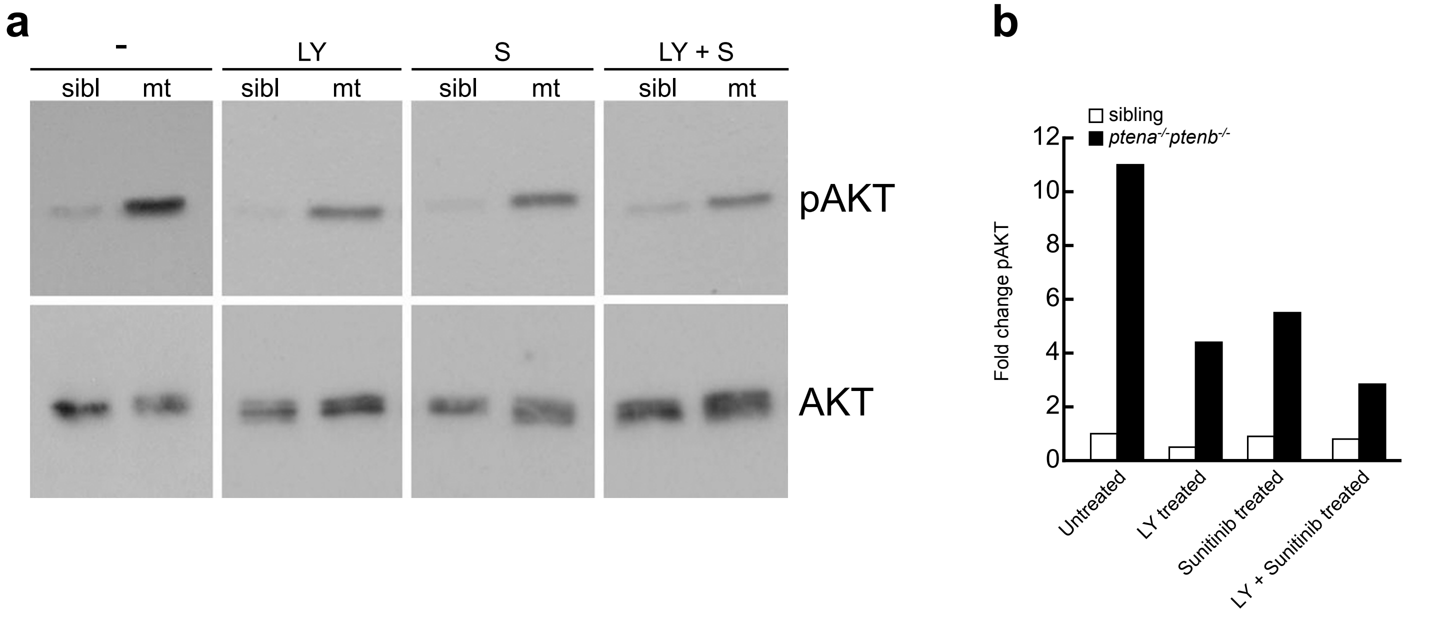

Suppl Figure 6. Combined treatment with suboptimal concentration of LY294002 and Sunitinib leads to mild down regulation of pAkt compared to non-treated Pten mutant embryos

(a) Sibling and *ptena-/-ptenb-/-* mutant embryos were left untreated (-), were treated with suboptimal concentration of 5 µM LY294002 (LY) or 5 µM Sunitinib (S) or both from 72 hpf onwards. Single embryos were lysed at 4 dpf and the protein from individual embryos was isolated. The proteins were run on a denaturing SDS-polyacrylamide gel and transferred to PVDF membranes. After blocking the blot was probed with phosphospecific pAkt antibody (directed against pSer473), stripped and probed with Akt-specific antibody as a loading control. The number of individual embryos that were analyzed is: sibl, 2; mt, 2; LY sibl, 4; LY mt, 4; S sibl, 6; S mt, 2; S + LY sibl, 12; S + LY mt, 9.Representative blots are depicted here. (b) Relative intensities of bands on immunoblots (pAkt level) was normalized against corresponding total Akt levels using the gel analyzer tool with Image J software and fold change was calculated using Excel.

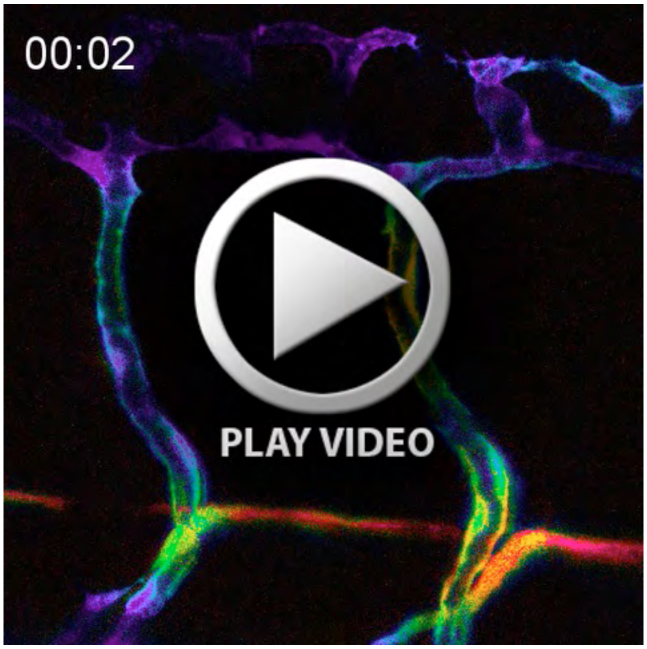

Suppl Movie 1. Endothelial cells of *pten* mutant siblings remain quiescent over time

Four dimensional imaging of siblings in *Tg(kdrl:eGFP)* was performed from 75 hpf onwards. Images were taken every 2 minutes, maximum projections of z-stacks (1 µm step size) were used to generate the time lapse movie. Z-planes are depicted with different colors. Anterior to the left, 40x with 2 zoom; time is indicated in hours and minutes.

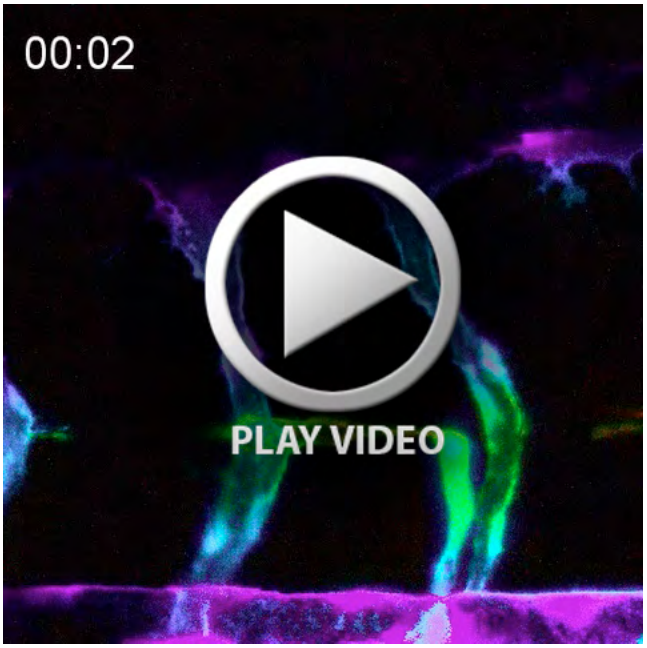

Suppl Movie 2. Endothelial cells in pten mutant embryos form filopodia over time
Four dimensional imaging of *ptena-/-ptenb-/-* mutants emrbyos in *Tg(kdrl:eGFP)* was performed from 75 hpf onwards. Images were taken every 2 minutes, maximum projections of z-stacks (1 µm step size) were used to generate the time lapse movie. Z-planes are depicted with different colors. Anterior to the left, 40x with 2 zoom, time is indicated in hours and minutes.
